# Genomic analysis of *Neisseria meningitidis* serogroup W causing invasive meningococcal disease in four travellers from Saudi Arabia, Italy, 2025

**DOI:** 10.2807/1560-7917.ES.2025.30.31.2500483

**Published:** 2025-08-07

**Authors:** Arianna Neri, Elsa Taviani, Federica Ferraro, Francesco Maraglino, Manuela Marra, Laura Daprai, Caterina Vocale, Elisa Vian, Anna Teresa Palamara, Paola Stefanelli

**Affiliations:** 1 Department of Infectious Diseases, Istituto Superiore di Sanità (ISS), Rome, Italy; 2 Italian Ministry of Health, Rome, Italy; 3 Servizio Grandi Strumentazioni e Core Facilities, Istituto Superiore di Sanità (ISS), Rome, Italy; 4 Microbiology and Virology Unit, Fondazione IRCCS Cà Granda Ospedale Maggiore Policlinico, Milan, Italy; 5 Microbiology Unit, IRCCS Azienda Ospedaliero-Universitaria di Bologna, Bologna, Italy; 6 UOC Microbiology Treviso Hospital, Department of specialist and laboratory medicine, AULSS 2, Treviso, Italy

**Keywords:** Invasive meningococcal disease, *Neisseria meningitidis*, Serogroup W, travel

## Abstract

Genomic analysis was performed on *Neisseria meningitidis* isolates from four cases of invasive meningococcal disease (IMD) identified in Italy after travel to Saudi Arabia in 2025. The cases were not epidemiologically related. The isolates from the cases were whole genome sequenced and belonged to serogroup W, sequence type (ST)-11, clonal complex (CC)11 and were susceptible to ceftriaxone, ciprofloxacin, penicillin G and rifampicin. Phylogenetic analysis grouped the four genomes with the Hajj strain sublineage.

Invasive meningococcal disease (IMD), caused by *Neisseria meningitidis*, is a severe, highly fatal acute bacterial infection manifesting as meningitis and/or sepsis, with rapid progression, requiring prompt medical support and antibiotic treatment. The highest incidence of IMD is observed among children, adolescents and young adults [1]. In April 2025, the World Health Organization (WHO) reported that on 13 March 2025, the National Focal Point (NFP) of Saudi Arabia notified WHO of 11 confirmed cases of *N. meningitidis* serogroup W (MenW) IMD in people attending Umrah in Saudi Arabia between 7 January and 12 March 2025 [2]. Here, we describe genomic analysis of MenW isolates from four persons who developed IMD after returning to Italy from a travel to Saudi Arabia.

## Case series

Four IMD cases, within the framework of National Surveillance System for Invasive Bacterial Diseases (IBD) (https://mabi.iss.it/; last access: 14 May 2025), coordinated by the Istituto Superiore di Sanità (ISS) and by the Italian Ministry of Health, were identified in patients after a travel to Saudi Arabia for pilgrimage or for business. No epidemiological link between the cases was identified. Two cases had meningitis and two had sepsis. None of them was vaccinated against MenW. All survived.

Local Italian clinical microbiology laboratories detected the presence of *N. meningitidis* by culture and/or PCR in cerebrospinal fluid (CSF) and/or blood samples and identified the isolates as belonging to serogroup W. Local public health authorities, in collaboration with the Ministry of Health, identified close contacts of the patients and prescribed chemoprophylaxis to them.

## Characterisation of *Neisseria meningitidis* isolates

Meningococcal isolates from the four cases were sent to the ISS for microbiological characterisation. Antimicrobial susceptibility to ceftriaxone, ciprofloxacin, penicillin G and rifampicin was determined with minimum inhibitory concentration (MIC) test strip methods (Liofilchem, Diagnostici, Italy) and clinical breakpoints according to the European Committee Antimicrobial Susceptibility Testing (EUCAST) (version 15.0) [3]. The isolates were susceptible to the tested antimicrobials (ceftriaxone: MIC < 0.125 mg/L, ciprofloxacin: MIC < 0.016 mg/L, penicillin G: MIC < 0.25 mg/L and rifampicin: MIC < 0.25 mg/L).

Whole genome sequencing (WGS) and core genome multilocus sequence typing (cgMLST) were performed on DNA extracted from the cultured isolates. Sequencing libraries were prepared using Illumina DNA Prep (Illumina, San Diego, the United States (US)) according to the manufacturer’s instructions. Assembled genomes were uploaded to the PubMLST database (https://pubmlst.org) [4] and communicated to European Centre for Disease Prevention and Control (ECDC).

All four sequenced MenW genomes belonged to sequence type (ST)-11, clonal complex (CC)11, finetype P1.5,2:F1–1 and harboured the fHbp peptide 9.

We compared the genomes using the BIGSdb Genome Comparator tool (https://bigsdb.readthedocs.io/en) (scheme *Neisseria meningitidis* cgMLST version 2) available on PubMLST [5,6]. The genomic comparison of 1,422 core genome loci was performed on eight genomes, five from 2025 (four obtained from the travellers described here and one from a sporadic case of MenW CC11 IMD) and three from 2024 (sporadic cases of MenW CC11 IMD) and submitted to PubMLST *Neisseria* database.

We also included five genomes used as references: the original Hajj strain (PubMLST ID2290), the endemic South African strain (PubMLST ID 21578), the Burkina Faso/North African strain (PubMLST ID 30087), the original United Kingdom (UK) strain (PubMLST ID30154) and the novel variant UK 2013 strain (PubMLST ID 30167) [7-9]. The resulting distance matrix was visualised as a Neighbour-Net network in Split Tree4 (version 4.13.1). As shown in Figure, the genomes branched into two areas: one with the reference genomes belonging to the Hajj strain sublineage and the second with the reference genomes belonging to the South American strain sublineage. The genomes of the four MenW CC11, here described, clustered with the genomes of the original Hajj strain, the Burkina Faso/North African strains and the endemic South African strain, distant from the remaining four MenW CC11 genomes identified in Italy in 2024–2025, the original UK strain and the new variant UK 2013 strain (Figure).

**Figure:**
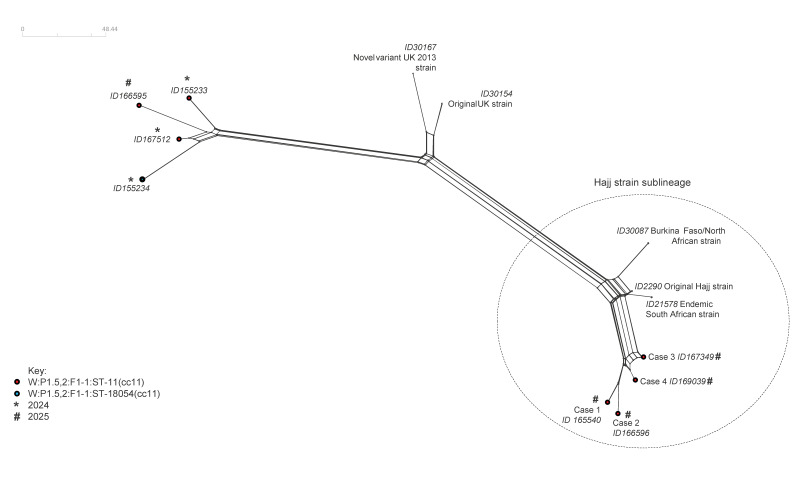
Neighbour-Net phylogenetic tree using core genome multilocus sequence typing of *Neisseria meningitidis* serogroup W (MenW) clonal complex 11 genomes, Italy (n = 8) and MenW reference genomes (n = 5)^a^

CC: clonal complex; cgMLST: core genome multilocus sequence typing; ID: identification code; ST: sequence type; UK: United Kingdom.

^a^ The genomes included were eight MenW CC11 genomes identified in Italy and five MenW reference genomes. The reference genomes were: original Hajj strain/ID2290; Burkina Faso/North African strain/ID30087; endemic South African strain/ID21578; original UK strain/ID30154; novel variant UK 2013 strain/ID30167). The Italian genomes were three from 2024 (ID155234, ID167512 and ID155233) and five from 2025: four obtained from the four travellers described in this paper (ID165540, ID166596, ID167349 and ID169039) and one from a sporadic case (ID166595).

The Italian genomes are marked with a coloured dot according to the ST. The asterisk refers to the isolation year of 2024; the gate symbol refers to the isolation year of 2025. Cases 1–4 related to travel to Saudi Arabia are also marked. The scale bar indicates the number of differences among the compared loci.

## Discussion

Invasive meningococcal disease caused by MenW in travellers returning from areas of mass gatherings, has led to subsequent intercontinental spread, favouring the emergence of MenW in geographical areas considered at low risk [10,11]. Important intercontinental outbreaks of IMD associated with Hajj occurred in 1987 (MenA), 2000 and 2001 (MenW), the latter leading to the emergence of MenW as a global public health issue [10-15].

The risk of meningococcal infections is considered low for pilgrims visiting the Hajj and Umrah areas in Saudi Arabia, who are vaccinated with the quadrivalent meningococcal vaccine, while the likelihood of infection is considered moderate for unvaccinated pilgrims visiting these sites [16].

In 2024, 12 cases of IMD caused by MenW were identified among people who had traveled to Saudi Arabia for Umrah pilgrimage: four cases in France, three in the UK and five in the US [17].

Overall, in 2022, 1,149 IMD cases were reported in the European Union and European Economic Area (EU/EEA) countries [18]. Serogroup B was most common (62%) among the cases with serogroup data available, whereas serogroup W accounted for 10%. A total of 1,347 cases of MenW infections were reported in 2018–2022 (https://atlas.ecdc.europa.eu/public/index.aspx).

In Italy, strains of MenW belonging to the Hajj-strain sublineage have already been identified in the past [19]. Here, we described the genomic analysis of four Hajj-related MenW isolates. We compared the four genomes with MenW CC11 genomes available in 2024–2025 in Italy. Phylogenetic analysis suggested that the four MenW isolates belonged to the Hajj sublineage; molecular analysis showed the presence of fhbp allele 9, also associated with the Hajj sublineage [17]. Several studies have described the heterogeneity of fHbp peptides among MenW ST11/CC11 isolates. Isolates of the South American sublineage from Chile, Argentina and Europe, as well as their descendants, the original UK strain and the 2013 UK strain, exhibited the fHbp peptide 22 [17], as the four non-Hajj MenW CC11 genomes collected in Italy in 2024–2025, included in the phylogenetic analysis of this report.

The risk for international spread of invasive meningococcall disease needs to be considered, as also highlighted by ECDC in a risk assessment published in 2024 [16]. In this risk assessment, ECDC recommended that EU/EEA public health authorities ensure travellers to the Hajj and Umrah areas in Saudi Arabia eligible for vaccination are counselled to receive the quadrivalent meningococcal vaccine (ACWY) at least 10 days before departure [16]. More recently, WHO has also recommended this and emphasised the importance of maintaining preparedness and surveillance throughout the year [2].

## Conclusion

Surveillance and genomic analysis allow identification of the presence of specific strains or emerging *Neisseria meningitidis* clones.

## Data Availability

Whole genome sequences are available on PuBMLST website (https://pubmlst.org).
